# Temporal refinement of 3D CNN semantic segmentations on 4D time-series of undersampled tomograms using hidden Markov models

**DOI:** 10.1038/s41598-021-02466-x

**Published:** 2021-12-02

**Authors:** Dimitrios Bellos, Mark Basham, Tony Pridmore, Andrew P. French

**Affiliations:** 1 School of Computer Science, University of Nottingham, Jubilee Campus, Nottingham, NG8 1BB UK; 2 Diamond Light Source Ltd, Harwell Science and Innovation Campus, Didcot, OX11 0DE UK; 3 Rosalind Franklin Institute, Harwell Campus, Didcot, OX11 0FA UK; 4 School of Biosciences, University of Nottingham, Sutton Bonington Campus, Loughborough, LE12 5RD UK

**Keywords:** Computer science, Scientific data, Software

## Abstract

Recently, several convolutional neural networks have been proposed not only for 2D images, but also for 3D and 4D volume segmentation. Nevertheless, due to the large data size of the latter, acquiring a sufficient amount of training annotations is much more strenuous than in 2D images. For 4D time-series tomograms, this is usually handled by segmenting the constituent tomograms independently through time with 3D convolutional neural networks. Inter-volume information is therefore not utilized, potentially leading to temporal incoherence. In this paper, we attempt to resolve this by proposing two hidden Markov model variants that refine 4D segmentation labels made by 3D convolutional neural networks working on each time point. Our models utilize not only inter-volume information, but also the prediction confidence generated by the 3D segmentation convolutional neural networks themselves. To the best of our knowledge, this is the first attempt to refine 4D segmentations made by 3D convolutional neural networks using hidden Markov models. During experiments we test our models, qualitatively, quantitatively and behaviourally, using prespecified segmentations. We demonstrate in the domain of time series tomograms which are typically undersampled to allow more frequent capture; a particularly challenging problem. Finally, our dataset and code is publicly available.

## Introduction

Over recent decades, multiple imaging approaches have been proposed in order to elucidate the hidden inner structure of many different systems captured in 3D volumes. X-ray computed tomography (CT)^1^, Magnetic Resonance Imaging (MRI)^2,3^, and Electron Microscopy (EM)^4,5^ are some of the methods that have been used for the collection of 3D information. At the same time, due to multiple technological advances, these methods have become progressively faster, allowing in recent years the collection of time-resolved volumes that form 3D movies of temporal events, or 4D datasets.

Across a plethora of applications, 4D imaging techniques are essential tools which provide researchers with detailed representations of temporal processes. However, the most difficult task in 3D or 4D studies is the *semantic segmentation* of the resulting datasets, which potentially requires many months of manual labelling. Fortunately, recent deep convolutional neural networks (CNNs)^6–11^ have shown an unprecedented improvement in 3D segmentation accuracy, where sufficient annotated data are available. Deep learning approaches achieve this by learning an end-to-end mapping between the input and the ground-truth, and later applying this learnt model to automatically segment new data.

Prior to the development of 4D (3D + time) techniques, approaches were proposed for the segmentation of videos (2D + time). Namely, Wang^12^ has proposed a watershed-based approach for tracking segmentations. Later, Price et al.^13^ have proposed a graph-based approach that propagates object segmentations in video frames. Recently, Hu et al.^14^ proposed a saliency-guided video approach that segments videos using a graph. Paul et al.^15^ also proposed a CNN-based video segmentation propagation approach. Nevertheless, recently by utilising video annotated datasets^16,17^, new approaches^18,19^ can achieve high segmentation accuracy. Regarding 4D data, multiple segmentation approaches have been proposed employing level-sets^20,21^, graph-cuts^22,23^ and Expectation Maximisation (EM) expanded to four dimensions using graphical models, like hidden Markov models (HMMs)^24–26^. While, some methods^20–24^ are able to segment 4D volumes without explicitly using 4D annotated data, they use prior knowledge about the data and their accuracy relies on high signal-to-noise ratio (SNR) data.

Furthermore recently, 4D segmentation deep learning approaches were proposed^27,28^. Their advantages is their robustness against low SNR data and easy application on data with different modalities. However, they do not utilize very deep networks, like 3D methods^7–9,11^ due to computational restraints. To overcome this, recent approaches^29,30^ utilize 2D convolutional LSTMs^31^. These approaches mainly work in 2D+time and their 4D output is attained by repeated application along the 3rd spatial dimension. Nevertheless, for most 4D deep learning segmentation techniques when applied on new objects of study, fully-annotated, 4D representative data rarely exist to train a solution.

**Figure 1:**
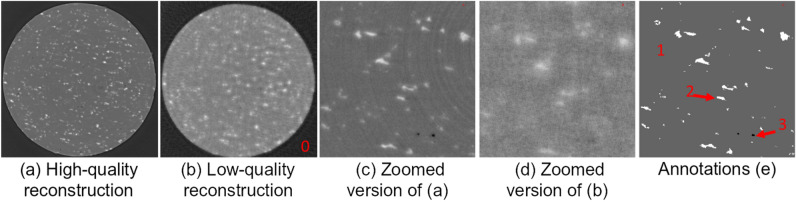
Cross section of our *real-world* dataset reconstructions and annotations. (**a**) Filtered Back-Projection (FBP)^36^ reconstruction from 3601 projections^37^ (these are cropped and centered around the metallic pin of Label 1), (**b**) Conjugate Gradient Least Squares (CGLS)^38^ reconstruction from 91 projections^39^ (these are cropped and centered around the metallic pin of Label 1), (**c**), (**d**) are zoomed versions of (**a**), (**b**) respectively and (**e**) are annotations for the same area^40^. Label 0 in (**b**) refers to the air outside the base-material, Label 1 in (**e**) refers to the base-material, Label 2 are the magnesium deposits and Label 3 are the gas pockets.

One such use-case, is the analysis of time-series sequences of micro X-ray CT scans. These time-series often consist of tomograms which are *undersampled* in terms of projections, due to the limited time available to scan of each tomogram. Each constituent tomogram of the time-series has to be collected quickly to allow for fast-occurring physical events, such as corrosion^32^, to be captured. By quickening the collection, satisfactory temporal-resolution is obtained for each time-series, but with a significant trade-off. The reduction of projections acquired creates an ill-posed reconstruction problem, and the resulting time-series tomograms are reconstructed with low SNR and imaging artifacts, (see Fig. 1b, d). To address this, we designed our Stacked-DenseUSeg^11^ that denoises and then segments these tomograms with improved accuracy using as a ground-truth highly-sampled *representative* tomograms (see Fig. 1a, c), more about them in “Results” section). Our method, though, is in principle 3D only, since it is extremely difficult to manually annotate the low SNR, time-series’ reconstructions (see Fig. 1b, d). For cases like this, where 4D annotations are insufficient for the development of true 4D segmentation approaches, expanding 3D CNN predictions to true 4D sequences is highly important. To address this, we propose the use of HMMs as *post-processing* methods to improve 3D segmentations generated independently through time.

In past research, HMMs have been combined with segmentation approaches, for image-video segmentation or detection^24–26^. However, this was not performed using contemporary and accurate segmentation approaches such as CNNs for 4D segmentation. A CNN has previously been combined with a HMM for number recognition^33^, but this approach is regarding classification in static images^34^, not video or 4D segmentation. To the best of our knowledge, this is the first attempt to refine multiple temporally consecutive 3D CNN segmentation labels with HMMs over time.

HMMs^35^, derive their name from their *hidden* states, modeled by a Markov chain. These hidden states form the model’s output prediction classes. Change between the hidden states through time is determined by transition probabilities, which are conditional probabilities of a hidden state’s value in the next time-step given its value in the current time-step. Additionally, at each time-step there is an input-observation with a value ranging from a set of possible *observable* states. These states are correlated to the hidden states through *emission* probabilities, meaning that specific observations of certain hidden states are more or less probable at each time-step. Finally, HMMs have starting probabilities for each of their hidden states. In essence, HMMs are Bayesian networks with the transition, emission and starting probabilities as their parameters. Specifically, our proposed HMMs use the temporally-consecutive 3D segmentations generated by our Stacked-DenseUSeg^11^ as input, and refine them to construct truly 4D segmentations by taking into account certain temporal properties. In summary:

1. We present a first HMM, named HMM-T, that uses temporal information from temporally-adjacent volume segmentations of a time-series. Our model combines empirically determined parameters and also parameters set by metrics from the 3D segmentation CNN to temporally refine these segmentations.
2. We present a second HMM, named HMM-TC, which unlike HMM-T also uses confidence information in the form of segmentation *probabilities* generated by the 3D CNN. Parameters are selected in the same manner as HMM-T.
3. We present experiments evaluating our HMM-T and HMM-TC. These provide qualitative demonstrations of their performance, test their behaviour under various conditions and also measure their accuracy in resolving temporal incoherence.

## Methods

**Figure 2:**
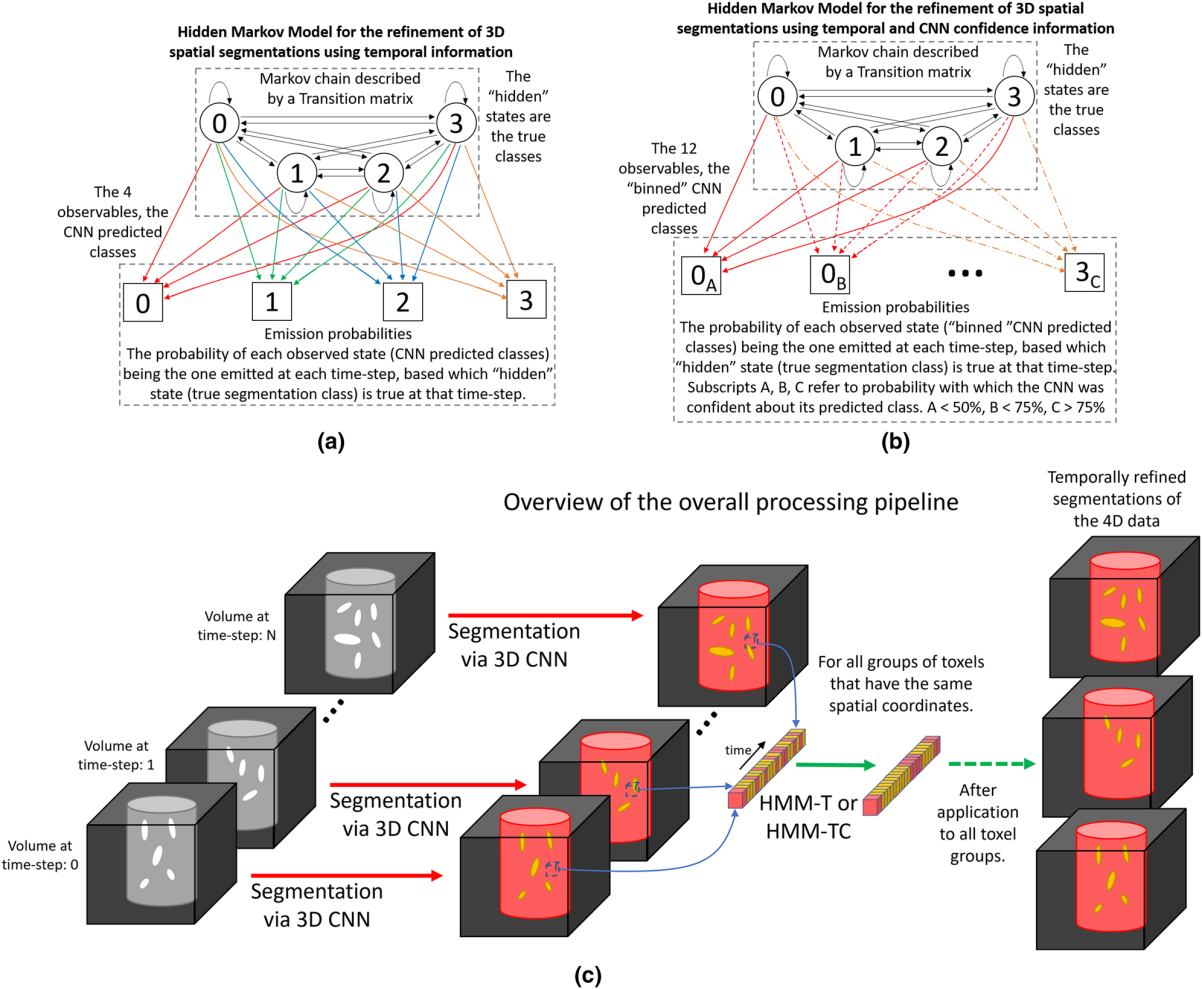
Hidden Markov models for the refinement of 3D segmentation predictions after the application of a 3D segmentation CNN on each step of a time-series. (**a**) A hidden Markov model, HMM-T that corrects the CNN predictions of a time-step using temporal information. (**b**) A hidden Markov model, HMM-TC, which in addition to temporal information also uses prediction probabilities generated by the CNN segmentation network alongside the 3D predictions. The black arrows represent transition probabilities from one class to the next through one time-step. In (**a**) the colored arrows represent the probabilities of each observed state (CNN predicted classes) being emitted at any given time-step based on the “hidden state” at that time-step (based on what is the correct segmentation class at that time-step). In (**b**) the same is true, but the observed states (CNN predicted classes) are also split to 3 levels (“bins”) of confidence. In either (**a**) or (**b**) the emission probabilities can be determined by the network’s confusion matrix during testing. Furthermore, in both (**a**) and (**b**) the transition probabilities are determined empirically. Finally (**c**) presents the overall processing pipeline. The constituent volumes in the 4D data are segmented independently through time and then HMM-T or HMM-TC refines all groups of toxels with the same spatial coordinates.

As explained in the previous Section, in this paper we are proposing two novel HMMs for the refinement of 4D data segmentation. Figure 2c offers an overview of the overall processing pipeline. Namely, every constituent tomogram-volume of the 4D data is segmented by a 3D segmentation network (Stacked-DenseUSeg^11^), independently through time. Then, every group of toxels (voxels of a time-step) with the same spatial indexes are processed independently by our HMMs models. This operation is valid since the 4D dataset is already aligned, and toxels with the same spatial indexes correspond to the same spatial coordinates in the physical sample.

In our first proposed model, HMM-T (a Hidden Markov Model using only Temporal information), the hidden states are the final segmentation predictions, and the CNN-predicted classes are the model’s observations. Furthermore in our second proposed model, HMM-TC (Hidden Markov Model using both Time and Confidence information), the observable states are not the predicted classes of a 3D segmentation network. Instead each of the CNN’s prediction-classes is further divided into three classes by the network’s confidence in them; in other words, the probability ascribed to its predictions. This is performed by dividing the CNN’s output classes into three different “bins”: low-confidence, mid-confidence and high-confidence, resulting in 3*N* new input classes, where *N* is the number of the original classes predicted by the CNN. For our models, the transition probabilities are empirically determined and their observation probabilities are derived from the confusion matrix of our Stacked-DenseUSeg during testing. They do not require training, as there are no high quality 4D annotations available to train our HMMs. Below we present our proposed models in further detail, as well the way in which their parameters are selected.

### HMM-T: hidden Markov model using temporal information from CNN segmentation predictions

As can be seen in Fig. 2a, HMM-T is composed of two sets of probabilities, transition probabilities $P(X_{t}|X_{t-1})$ that determine the probabilities of (hidden) states transitioning from one to another through time, and emission probabilities $P(X_{t}|E_{t})$ that determine the probabilities of each (hidden) state being “emitted’ for each of the observable states.

The $P(X_{t}|X_{t-1})$ probabilities that denote the transition probabilities from each (hidden) state to another through time, form what is know as a Markov chain. Thus, they can be expressed using a $N \times N$ matrix $\varvec{T}$ (see Supplementary Matrix S1), named the transition matrix, where *N* is the number of segmentation classes. On the other hand, the $P(X_{t}|E_{t})$ probabilities express the probability a segmentation class can be predicted as any other by a segmentation network, forming a $N \times M$ matrix $\varvec{O_1}$ (see Supplementary Matrix S5), named the emission matrix. For the simple case, where the predicted classes from a segmentation network are the same in number as the (hidden) states (the segmentation class inferred by the HMM), then $M=N$ and $\varvec{O_1}$ is also $N \times N$. The other important parameters in HMM models, apart from the transition and observation probabilities, are the starting probabilities of each of the (hidden) states. They express the probabilities for each state to appear at $t=0$ (assuming indexing starts with 0, $t=0,1,2,\ldots$), compensating for the absence of a previous state (there is no $X_{-1}$) and so the transition probabilities can not be used. These probabilities can be expressed by a vector $\varvec{S}$ with size $1 \times N$. Provided that all HMM parameters are determined, using a forward prediction algorithm like the Maximum a posteriori estimation (MAP) or the Viterbi algorithm^41^, the segmentation classes of each group of toxels with the same spatial coordinates can be determined. Since our proposed hidden Markov models use only temporal information from previous and subsequent segmentation predictions, we will refer to it as HMM-T in the following sections.

### HMM-TC: hidden Markov model using temporal information from CNN segmentation predictions and its confidence for them

As can be seen in Fig. 2b, in HMM-TC the observable states (CNN predicted classes) are further split based on the CNN’s confidence of the relevant classes (i.e. the CNN’s segmentation probability). Since HMM-TC uses the probability of the leading class for each of the CNNs predictions, this can be within the range $(\frac{1}{N}, 1)$, where *N* is the number of the segmentation classes. This is because, in case the network is not confident about its prediction, the probability distribution between the different classes is uniform and so the $P(X=leading~class) \rightarrow \frac{1}{N}$. On the other hand, when it is very confident regarding its prediction $P(X=leading~class) \rightarrow 1$. In order to introduce the confidence information to the emission matrix $\varvec{O_2}$, the network’s segmentation probability for the leading class is quantized based on a number of “bins” that equally split the range $(\frac{1}{N}, 1)$. Then each original observable states (same as in HMM-T) is split to new observable state that are equal to the number of “bins”. For instance using *b* “bins” to split the $(\frac{1}{N}, 1)$ range and *M* observable states the total of the new observable states is *bM*. Figure 2b depicts the case where 4 observable classes (the network’s prediction classes) are split by 3 “bins” ($(25\%,50\%)$, $(50\%,75\%)$, $(75\%,100\%)$) to create 12 new observable states. We also refer to these 3 “bins” as low-confidence, mid-confidence, high-confidence.

### Selection of hidden Markov model parameters for the segmentation of time-series of tomogram reconstructions

As described in “Introduction” section, time-series of X-ray micro CTs are collections of multiple tomograms that capture temporal events. These tomograms are undersampled, meaning that their signal-to-noise ration is small, making it very difficult to manually segment their reconstructions. Thankfully, with iterative application of our 3D segmentation network Stacked-DenseUSeg^11^, the individual tomograms in a time-series can be segmented. However, our approach is 3D and inter-volume (inter-tomogram) information is not utilized. Sadly, it is not possible to create a supervised approach, as for our Stacked-DenseUSeg network, directly for 4D data without high quality 4D annotations. Such annotations, with the same level of quality as annotations of fully-sampled tomograms used to train Stacked-DenseUSeg, are practically impossible to acquire due to the extremely low level of the SNR of the mid-time-series’ tomogram reconstructions. Segmentation of other 4D datasets with deep learning may face similar issues, if there are other reasons that prevent the collection of a sufficient amount of good quality 4D annotations. Therefore, it is important to develop segmentation *refining* techniques for 3D segmentation CNNs that use inter-volume information and do not require full 4D annotated data.

Based on the above scenario, the proposed hidden Markov models presented here are used in an unsupervised manner that do not require 4D annotated data. Values for their transition probabilities, described by matrix $\varvec{T}$, are empirically determined based on the expected physical behaviour of our datasets^32^. For example, we selected the transition probability of Magnesium to Gas Pockets to be approximately 10% which reflects an expectation of real-world behaviour, and we also introduce some non-zero transition probabilities between other classes. These non-zero transitions are in place for very rare situations where the transition between two classes is a product of errors such as the misalignment between the tomograms in the time-series, or other physical events outside of experimental control, such as thermal expansions and contractions. The final transition matrix $\varvec{T}$ for the HMM-T and HMM-TC models is presented in the Supplementary Information as Supplementary Matrix S1. In order to evaluate the accuracy of our proposed HMM models that use the Supplementary Matrix S1 as their transition matrix, two more variations of the HMM-T that use different matrices will be tested in the following “Results” section. The first is HMM-T-naive (transition matrix in the Supplementary Matrix S2) and the second one is the HMM-T-naive-stable (transition matrix in Supplementary Matrix S3). The HMM-T-naive considers that all transition from one class to another, including the current class, is equally probable. HMM-T-naive-stable considers that in every step there is 50% chance for a transition to a different class, uniformly distributed over the classes different from the current one. Both HMM-T variations provide a baseline against HMM-T (that uses Supplementary Matrix S1 as its transition matrix), in order to evaluate the accuracy of HMM-T’s transition matrix to resolve temporal incoherence, it being empirically determined based on the expected physical behaviour of our datasets^32^. Regarding the emission matrix $\varvec{O_1}$, the confusion matrix during the testing of the segmentation CNN is used (see Supplementary Matrix S4). This is because the confusion matrix can provide a clear picture regarding emission distributions, which are the probabilities of each class being predicted by the network as any other class. Namely, every row of the Confusion Matrix is divided by the sum of its cells and the emission matrix for the HMM-T is presented in the Supplementary Information as Supplementary Matrix S5. As can be seen in the bold text in Supplementary Matrix S5, for each of the observable states which are the predicted classes from the CNN, the state/class that has the higher probability to emit them is the same as the observable states.

Moving on to the HMM-TC model, the emission matrix is also calculated via the confusion matrix of the CNN during testing. During the network’s testing, the calculation of the confusion matrix is coded to distinguish between new observable states and produce a rectangular confusion matrix (see Supplementary Matrix S6). Supplementary Information presents the resulting Emission Matrix $\varvec{O_2}$ as the Supplementary Matrix S7. As can be seen, the observable states sometimes are emitted with higher probability by classes that are not intuitively expected to emit them. For example the Mid-Confidence Aluminium label is “emitted” with higher probability by ground-truth Gas Pocket pixels. This is because the probability of “emitting” any other label other than Gas Pocket by a ground-truth Gas Pocket voxel (voxels - not toxels - because Stacked-DenseUseg’s testing ground-truth is 3D) is estimated as the ratio of Gas Pocket voxels erroneously recognized by our deep-net as any other label to all testing ground-truth Gas Pocket voxels. As the dataset contains much less voxels labeled as Gas Pocket than other classes, an occasional misclassification affects the estimate of emission probabilities for the Gas Pocket hidden state much more than for other hidden states. Namely, the denominator (the sum of all occurring Gas Pocket voxels) is significantly smaller than the denominator in other states/classes (the sum of all occurring Air, Aluminium, etc. voxels) because Gas Pockets are more rare. Naturally, the high probabilities are also intensified by the numerators, namely the number of Gas Pocket voxels predicted as low, mid or high confidence voxels of any other class. The high misclassification of “difficult” classes (that are comprised of spatially small objects/instances, which have intricate shapes and are therefore difficult to accurately segment by a 3D CNN) such as Magnesium and Gas Pockets, is due to the ground-truths used during the CNN’s testing. Namely, these ground-truths are high-quality annotations from a fully-sampled tomogram and they are very detailed. Due to the downsampling of projections, though (which during the training of a 3D CNN it is artificially preformed to the fully-sampled tomogram to create the network’s input), details of these spatially small classes are lost in the input. During the CNN’s training, this is not an issue since the network is pressured to not only segment easily distinguishable objects in the input, but to also potentially restore missing information, increasing its accuracy further. Obviously, all missing information between the undersampled and fully-sampled version of the input can not be restored, and this is true even for an infinitely large and deep CNN. In general, there is a level of information that can not be restored after the downsampling of projections and the remaining information is not sufficient to enable the prediction and restoration of the missing information.

As mentioned earlier, there are a number of network misclassifications that are not caused by the inability to segment accurately, but rather due to the extreme information loss, which cannot be restored by any means. However, for the calculation of the confusion matrix during testing (and indirectly also the observation matrix), a highly detailed ground-truth is used, which are the manual annotations of the high SNR reconstruction of the representative highly-sampled tomogram. Therefore, the aforementioned misclassified voxels are considered during the calculation of the confusion matrix and also the observation matrix (some Gas Pockets are predicted as Mid-Confidence Aluminium), when they should not since there is not sufficient information in the provided input to the network to restore missing information. For this reason, the earlier probabilities of the emission matrix are manually adjusted to account for the existence of these “information-loss-caused” misclassified toxels. Specifically, emission probabilities are modified so that any low, mid or high confidence predicted class is more likely emitted by the class with the same title. For instance using the previous example, parameters are adjusted so that Low-Confidence Air and Magnesium are more likely “emitted” respectively by Air and Magnesium. Supplementary Information presents the resulting adjusted matrix $\varvec{O}_{2}^{*}$ as Supplementary Matrix S8. One advantage of models such as HMMs is the human readability of such matrices, and the ability to adjust by hand if required.

Finally regarding the starting probabilities, there is not a reason that makes any state/class more plausible at $t=0$. While some states/classes are more frequent due to their relative population in the tomogram reconstructions, this is a class imbalance true for every time-step and not specifically for $t=0$. For this reason, we elected starting probabilities that have a uniform distribution between the different states/classes. Finally we selected the Viterbi algorithm^41^ to refine the input predictions. Additionally, to speed up the process we use a computational shortcut. Namely, for groups of toxels with the same spatial coordinates where the observable state is the same through time (something very likely in large 4D datasets with temporal events transpiring in localised regions only) we assign the same class to these toxels without using the hidden Markov models. Specifically, we detect in the observable states’s corresponding column in the Emission matrix what class emits this observable state with the highest probability. Then we assign the classes to these toxels. This shortcut allows skipping the Viterbi algorithm for numerous toxels, saving a great deal of computational effort.

## Results

In this paper, we utilize an existing tomogram time-series (4D dataset)^42^, where its constituent tomograms have individually been segmented by our CNN approach^11^. The resulting segmentations are then refined by our proposed methods, which use one of two HMM configurations. Our tomogram time-series was collected at the Diamond Light Source I13-2 beamline^43^. It depicts a droplet of salt-water on top of a 500-micron aluminium pin with magnesium deposits and it is a part of a study to measure the corrosion triggered by the salt-water droplet over time^32^. It is comprised of 21 low SNR tomograms, and their reconstructions have a $2160\times 2560\times 2560$ voxel resolution. In^42^ the tomograms are cropped to $1710\times 1310\times 1310$ voxel resolution to reduce their memory footprint. The cropped version depicts the entire physical sample, excluding only background air voxels. The data used to train Stacked-DenseUSeg^11^ are also available online^37,39,40^, as well as the code^44^ including both the HMM models and Stacked-DenseUSeg. There are four segmentation classes: the air outside the base-material (Label 0 in Fig. 1c), the base-material (mostly aluminium) (Label 1 in Fig. 1e), the magnesium deposits within the base-material (Label 2 in Fig. 1e) and lastly the gas pockets within the base-material (Label 3 in Fig. 1e). For the training of our Stacked-DenseUSeg a single highly-sampled tomogram is used. This tomogram is captured at the end of tomogram time-series collection, after the termination of any temporal events. Since it is captured outside of the critical time-window, it is collected slowly to obtain a high SNR reconstruction and it has too a $2160\times 2560\times 2560$ voxel resolution. As mentioned in^11^, this tomogram was accurately annotated and split into multiple non-overlapping $64^3$ cube-inputs. From those, 70% (3723 cubes) are used for training, 20% (1065 cubes) for testing and 10% (532 cubes) for validation. The trained network then used to segment the time-series tomograms independently though time.

Naturally, the application of the 3D CNN independently through time ignores the relationships between consecutive time-steps, and we hypothesize that additional improvement can be attained by comparing *temporally adjacent* predictions, since there are physical rules for the temporal progression of the studied processes. Due to the physical properties of the temporal events, there are expected and unexpected outcomes. For instance, the corrosion occurring in our dataset postulates that traces of magnesium within the sample would naturally corrode and disappear leaving behind gas pockets (class 3). On the other hand, background air (class 0) is not expected to change to something else. Also to consider, the CNN’s segmentation results have a different level of accuracy depending on the predicted class. Together then, there are certain transition probabilities for the CNN segmentations through time as well as emission probabilities for them. This behaviour can be modelled using our proposed HMMs (see “Methods” section). This section will present the results of qualitative, behavioural and quantitative experiments using our HMM-T and HMM-TC models.

As of now, most proposed 4D segmentation approaches which do not require annotated 4D data, directly segment 4D data, without first segmenting 3D volumes independently through time and then refining them. Additionally, they rely on high SNR data to achieve high accuracy. Without the utilization of annotated or high SNR 4D data, these approaches are unfit to handle the low SNR of time-series of undersampled tomograms. Because of this, valid comparison against them, even though desirable, cannot be performed. For these reasons, instead the results of the quantitative experiments presented in this section will be compared against two variants of HMM-T: HMM-T-naive and HMM-T-naive-stable, described in “Selection of hidden Markov model parameters for the segmentation of time-series of tomogram reconstructions” section, that employ unsophisticated transition matrices that are not based on any prior knowledge for the expected physical behaviour of our datasets. Furthermore, we will also compare HMM-T and HMM-TC against a 1D median filter with window size of 5 time-steps, as a way of comparing to a simplistic post-processing approach.

### Qualitative assessment of the HMM predictions

**Figure 3:**
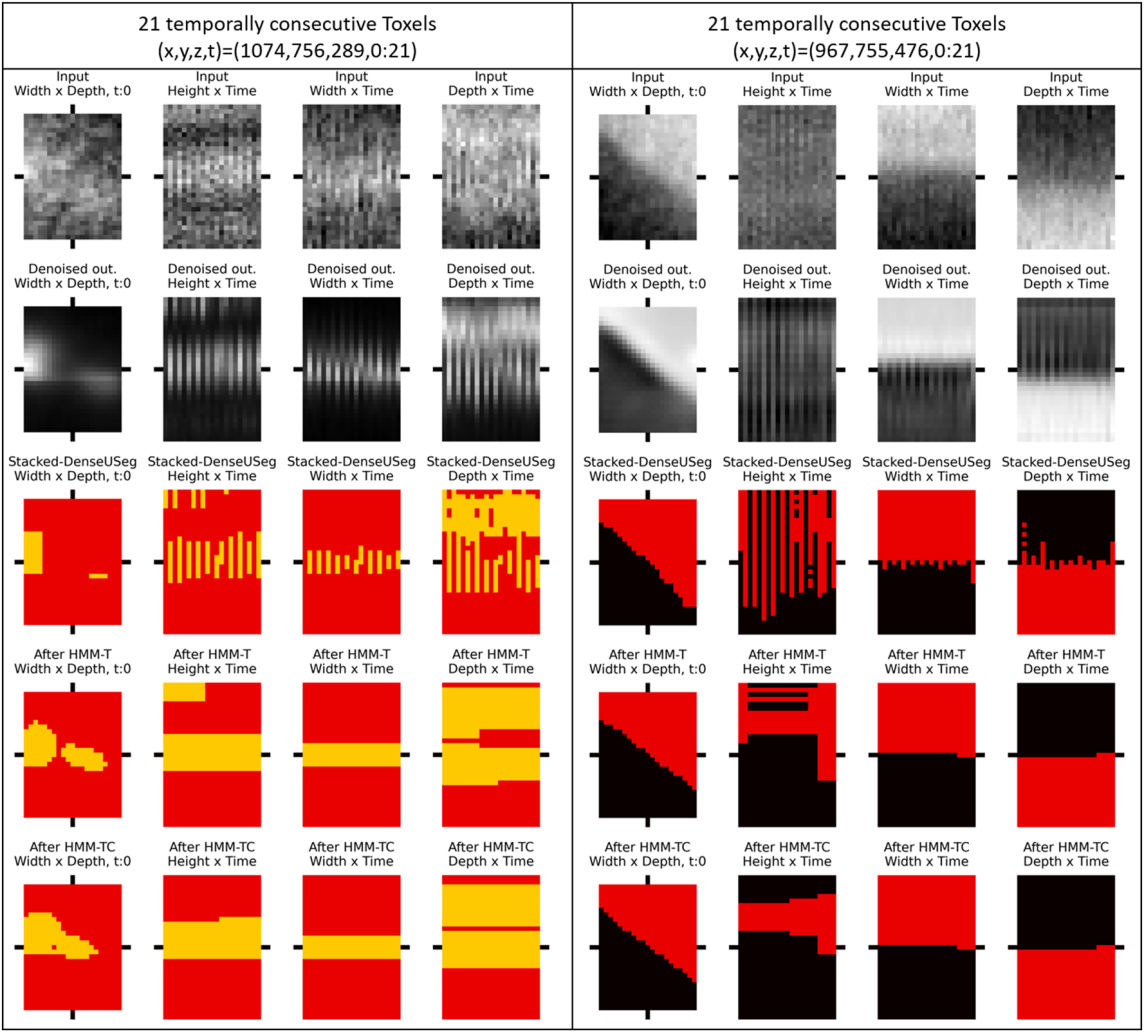
Comparison of segmentations of 2 groups of temporally consecutive toxels and their neighboring toxels (both spatially and temporally). The subfigures display predictions through the Width–Depth plane (*xz*-*plane*), Height–Time plane (*yt*-*plane*), Width–Time plane (*xt*-*plane*) and Depth–Time plane (*zt*-*plane*) respectively, after the use of Stacked-DenseUSeg (**3rd row**), Stacked-DenseUSeg + HMM-T (**4th row**) and Stacked-DenseUSeg + HMM-TC (**5th row**). The black bars indicate that the toxels in question are the ones in the middle and the black crosshair point to the central toxel that belongs to first time-step ($t=0$). From the subfigures, it can be deduced that the HMM models solve temporal incoherence in the Stacked-DenseUSeg’s predictions. HMM-TC seems that it does not allow many transitions, potentially due to the utilization of Stacked-DenseUSeg’s prediction confidence in its emission matrix.

One of the first things examined in this Section are the visible effects of our HMM models on the 4D segmentations generated by time-independent application of a 3D CNN approach^11^ to a time-series of tomograms. For this, we choose to focus on certain interesting *toxels* that their class prediction alternates frequently through time. These feature indicate class transitions which are physically improbable. Examples of these toxels can be seen in Fig. 3. In the Figure, we present two examples of “edge-toxels”, meaning that they are close to a spatial edge of a segmentation instance. As can be seen with the help of spatially neighboring toxels, their class predictions through time alternate between two classes. Due to their proximity to the spatial edge, the segmentation probabilities of these toxels are close to 50–50 between two classes, and so random noise may dictate the final prediction, which differs at each time-step. However, our HMM models regulate the noisy predictions and allow transition between the classes to happen smoothly and in a rational manner. Furthermore, as seen in the Figure, the HMM-TC refined predictions contain less such transitions, which may be attributed to the incorporated CNN confidence information, allowing it to offer even more informed output predictions.

A broader view of the segmentation refinement can be obtained from Supplementary Fig. S1. As can be seen, the refined predictions of our HMM models remain relatively similar to our Stacked-DenseUSeg’s predictions. One indication of their refinement can be seen in mark $\varvec{I_1}$ in Supplementary Fig. S1, where the earlier mentioned “edge-toxels” (seen as “jagged” edges in the Stacked-DenseUSeg’s predictions) now belong to the same class through time.

### Behavioural experiments using HMM-T

In an effort to further examine the behaviour of our HMM models we assigned specific toxels to certain classes through time before running the HMM. This allowed us to simulate various scenarios of segmentations offered by a CNN. We only used the simpler HMM-T foundation model in these tests, as we wished to explore only the behaviour of our HMMs with certain (100% confident) segmentation labels (versus HMM-TC which includes fractional confidence measures from a real CNN output).

**Figure 4:**
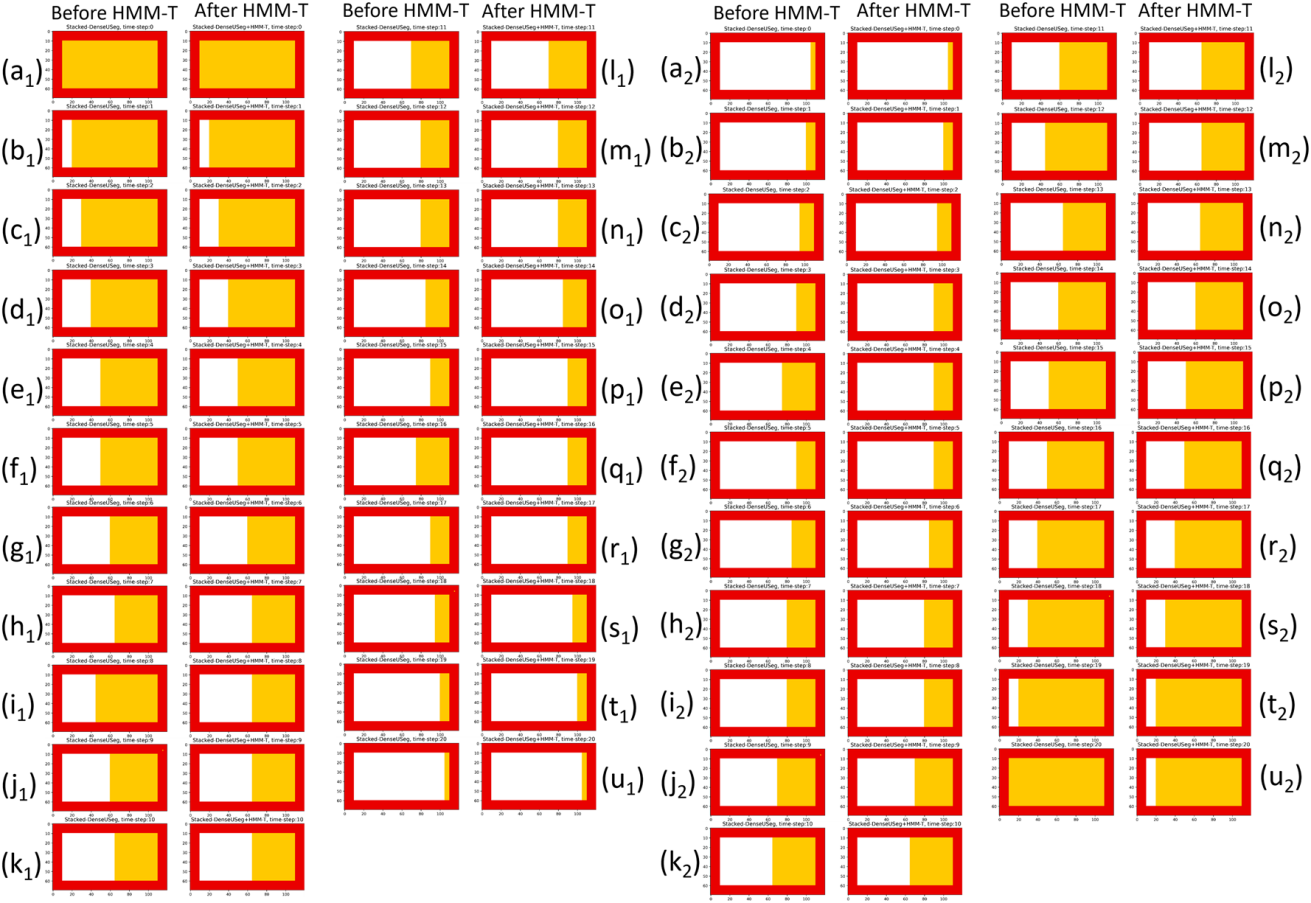
Behavioral tests of the HMM-T for the prediction of the Magnesium (class = 2, yellow) turning into Gas Pockets (class = 3, white) over time (**a**$_1$–**u**$_1$) and vice versa (**a**$_2$–**u**$_2$). (**a**–**u**) in both test refer to the time-steps 0–20 respectively. The slice displayed is the 740th (out of 1710) of the height dimension (*y*-*axis*), for the ROI [696:1304, 10:522] (*xz*-*plane*). The left-hand side of the figure shows an expected transition direction, but with noisy (unexpected) input at (**i**$_1$) and (**q**$_1$). As can be seen in these noisy positions, the gas pocket prediction (white) remained in place, rather than following the input data, which is desired since magnesium (yellow) has a high probability to turn into gas pockets (white), but not the opposite way around. In the right-hand side of the figure, the opposite transition is simulated; as can be seen in (**e**$_2$), (**l**$_2$–**n**$_2$), (**u**$_2$) a number of gas pocket predictions (white) persisted between time-steps, which is expected since gas pockets (white) have low probability to turn into magnesium (yellow). Specifically (**t**$_2$–**u**$_2$) shows that without a follow-up observation to provide support for what (**u**$_2$) displays (no Gas Pockets), HMM-T kept the predictions of the previous time step. However it also shows that the non-zero transition probabilities do allow reverse transitions where the observation data is overwhelmingly confirmatory.

First then, we investigate the effects of our empirically determined transition probabilities (see Supplementary Matrix S1). For this experiment, multiple toxels are deliberately assigned to either one of two classes, Magnesium or Gas Pockets, and over time they transition between them. The elected transition probabilities favour Magnesium to Gas Pockets class changes, and not the opposite direction. This is based on physical properties of the sample, since according to the underlying chemical reactions, magnesium corrodes and then escapes as gas, leaving in its place gas pockets. As can be seen in Fig. 4a$_1$–u$_1$, toxels of Magnesium (yellow) are slowly changing into Gas Pocket. The geometry of the assigned toxels is not significant since the HMM model process toxels through time independently as a 1D sequence. As is shown in (i$_1$) and (q$_1$), where toxels act in a way that is physically impossible and transition from gas pockets to magnesium, HMM-T corrects this and keeps the toxels assigned as gas pockets. As this transition is physically impossible, and occurs only infrequently, the HMM has corrected what could be a prediction error (which may occur with time-independent application of 3D CNNs on 4D data).

We also tested HMM-T behaviour with the reverse class assignments through time, as shown in Fig. 4a$_2$–u$_2$. For this we test the model’s behaviour under repeated *unexpected* observations despite their being unlikely. Due to the high emission probabilities (see Supplementary Matrix S5) as a result of Stacked-DenseUSeg’s high prediction accuracy, and due to the abundance of observations (see Supplementary Matrix S1), HMM-T eventually permits these “erroneous” transitions. Additionally, based on Stacked-DenseUSeg’s different accuracy per class, Magnesium predictions indicate Magnesium with a higher probability than Gas Pockets predictions indicate Gas Pockets. This means that the model is more inclined to trust Magnesium predictions than Gas Pocket predictions. Nevertheless, in (e$_2$), (l$_2$–n$_2$), (u$_2$), the effort of our model to prevent these transitions is visible. Especially in (l$_2$–m$_2$), they are delayed/prevented since there is insufficient evidence for them at those time-steps. Finally, in u$_2$, the Magnesium predictions are not enough for HMM-T to not allow the transition. It seems that more than one consecutive Magnesium prediction is needed for the HMM-T to permit the transition.

In conclusion, while we want our HMMs to refine the segmentations and temporal incoherence (left-hand side of Fig. 4), we do not want ito alter data when there is an overwhelming evidence of improbable predictions (right-hand side of Fig. 4). This is because there may be other 4D datasets where certain class transition may only be slightly less likely, and not physically improbable. It is important not to “muffle” certain temporal events, just by the premise that they are unlikely. Unexpected transitions may also exist due to temporally misaligned tomograms or errors in the 3D CNN design. Our HMMs, while designed to resolve temporal incoherence, should not correct these types of errors on all occasions, but allow the user to locate them.

As a final experiment, we also tried replacing *all* predictions in a single time-step with just class 1, Aluminium. After the application of the HMM-T, our model was able to recover almost of the missing information (see visual results in Supplementary Fig. S2). This simulates a potential data loss and recovery scenario. Naturally, this task is easier with 4D with high temporal-resolution, as neighbouring time-steps are closer in appearance and so it is easier to predict missing information between them. This experiment clearly indicates the benefits of using inter-volume information for the refinement of 4D segmentations from a 3D CNN.

### Quantitative experiments

Next, we will attempt to quantitatively measure our models’ segmentation accuracy. Preferably this can be tested using sufficient and accurate 4D segmentation annotations. However, our time-series tomograms are *undersampled*, meaning that it is practically impossible to accurately segment them fully manually. Due to their low SNR, it is extremely difficult for human annotators to discern exact edges in them, let alone restore missing information due to the downsampling of projections.

Given this situation, there are two available options for evaluating segmentation accuracy. The first is to measure it an area of the sample where temporal events are *unlikely* to have occurred due to physical expectations of the studied system. For this sub-volume through time, it would then be valid to consider as a ground-truth, at any time-step, the annotations from a highly-sampled tomogram that images the system either before or after the time-series. The second option would be to manually annotate a small sub-volume through time where temporal changes are likely to have occurred. Obviously, due to the difficulties mentioned earlier, these annotations may contain errors. Nevertheless, it would be inappropriate not to test our models in critical areas where temporal phenomena occur, so despite its shortcomings, the second method will also be employed.

First then, we test in the *static* region using the high-sampled tomogram annotations as ground-truth in all time-steps. Table 1a presents the Intersection over Union (IoU) achieved for each class and the mean IoU. For this comparison, 10 slices (slices from height 1210–1220) through the 21 tomogram time-series ($10\times 21=210$ images) are used from a static region of the sample. Temporal events in our datasets are caused by a droplet of salt-water triggering corrosion, so areas of the sample sufficiently far from this region are not expected to change over time. For these areas our HMMs are expected to resolve mainly noisy boundaries, as demonstrated in Fig. 3.

From the Table 1a we see that HMM-T is the best performing model, but HMM-TC is also better than HMM-T-naive, HMM-T-naive-stable and the median filter. In particular, both HMM-T and HMM-TC improve the IoU score of Magnesium and Gas Pockets noticeably. These classes are regarding small segmentation instances that, due to noise, are not segmented with consistent accuracy by Stacked-DenseUSeg through time. Based on these facts, HMM-T and HMM-TC using inter-tomogram in Stacked-DenseUSeg’s segmentations and knowledge about the physical system communicated by their empirically determined transition matrix, better improve the IOU score for Magnesium and Gas Pockets compared to the other methods. Regarding the class of Air and Aluminium, it can be seen that all methods offer almost identical IoU, as a result HMM-T and HMM-TC offer overall slightly better the mean IoU score. Finally, as might be expected HMM-T-naive has the same IoU metrics as the unrefined segmentations in Table 1a–c. By considering the transition between classes equally probable at each time-step, any possible sequence of classes is considered temporally coherent and so there no need for refinement.

**Figure 5:**
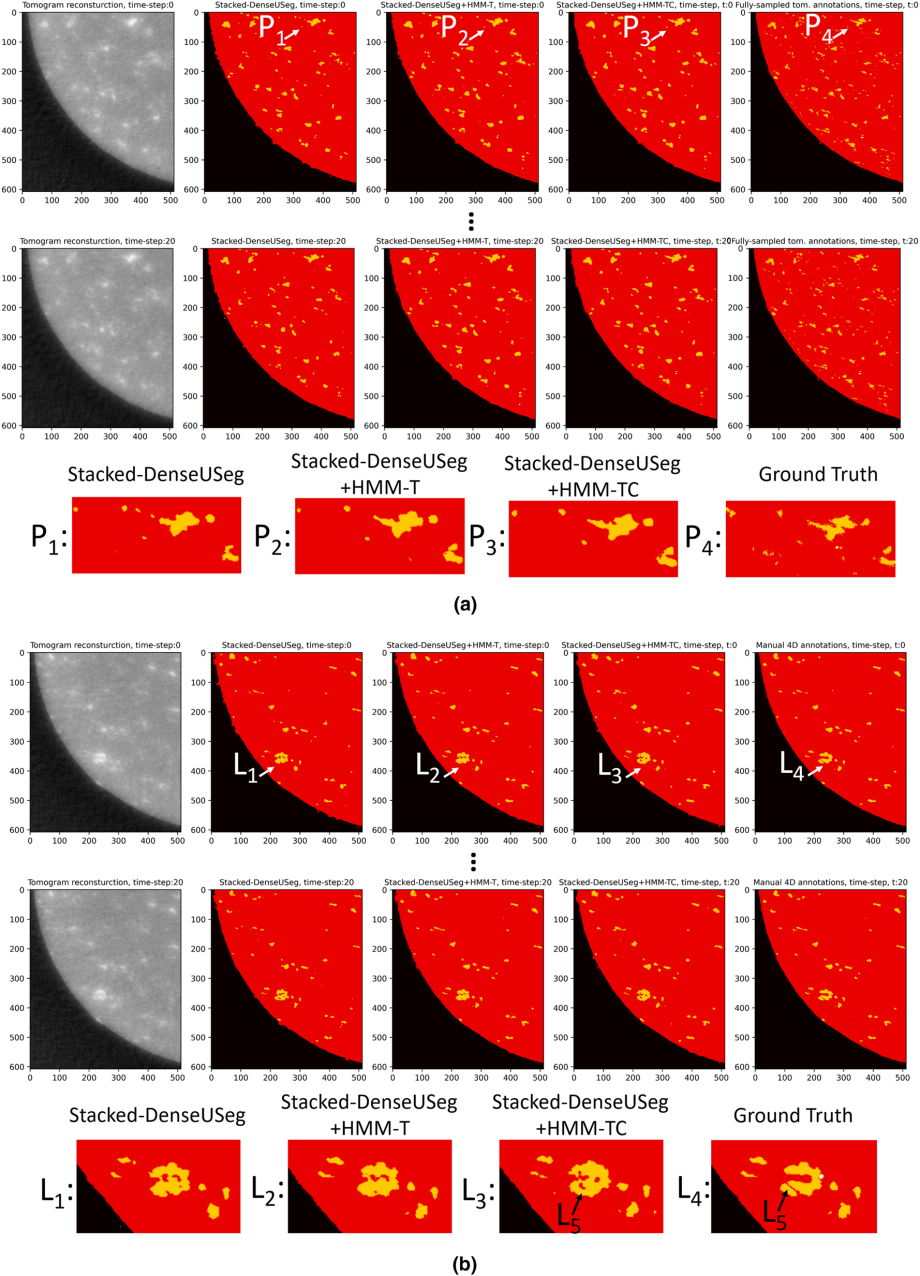
Comparison of segmentation predictions in a time-series of tomograms after the use of Stacked-DenseUSeg (No HMM), Stacked-DenseUSeg + HMM-T, Stacked-DenseUSeg + HMM-TC. (**a**), comparison against the annotations from the corresponding representative fully-sampled tomogram to the time-series. For the comparison, 10 slices (slices 1210–1220 height-wise) are used at the bottom of the imaged sample in the time-series. This is to ensure that temporal events did not take place and that the annotations from the representative fully-sampled tomogram of the time-series are valid though time. The figure displays the 1210th slice out of 1710 ($y-axis$) and for the ROI [696:1304, 10:522] ($xz{-}plane$). (**b**) comparison against our manual 4D annotations. For the comparison 2 top slices (slices 740–741 height-wise) are used in the imaged sample of the time-series. This is to help ensure that temporal events occurred in these areas and that the comparison would provide some evidence about the temporal behaviour of our HMM models for a dynamic system. The figure displays the 740th slice out of 1710 ($y{-}axis$) and for the ROI [696:1304, 10:522] ($xz{-}plane$). In both (**a**) and (**b**), Marks $\varvec{P_1}$–$\varvec{P_4}$, $\varvec{L_1}$–$\varvec{L_5}$, and their zoomed versions provide a qualitative indication of the advantages of the HMM models.

This is evidence for the advantage of HMMs for the refinement of temporally consecutive 3D segmentations. The reason behind the elevated performance of HMM-T over HMM-TC can potentially be attributed to the complexity of choosing suitable values for HMM-TC’s observation matrix (explained in depth in “Selection of hidden Markov model parameters for the segmentation of time-series of tomogram reconstructions” section), which we found had to be adjusted manually to produce suitable results. In Fig. 5a the resulting annotations are presented. As can be seen in marks $\varvec{P_1}$–$\varvec{P_4}$ and the zoomed images, the improvement that the HMM models offer is quite noticeable, especially in the “tail” area in the zoomed images. Here, arguably the HMM-TC model’s visual performance appears to be slightly better, due to it resembling more closely the high-sampled tomogram’s annotations.

After the use of annotations from a highly-sampled tomogram in a *static* sub-volume, we continue with comparisons using our manual 4D annotations as ground-truth in a dynamically-changing section. For this test we carefully annotated 6 slices (slices from height 740–742 and 747–749) through the 21 tomogram time-series ($6{\times }21{=}126$ images), which we hoped would be most prone to corrosion, and hence allow us to evaluate our models in areas where temporal phenomena occurred. These annotations are available online^45^. Unavoidably, there may be some errors and bias incorporated in the annotations, however, as previously mentioned there is not an alternative for measuring the segmentation accuracy in these areas. For the generation of the annotations we used HMM-T predictions as a template, and then refined the labels manually. As can be seen in Table 1b, c, HMM-T is the best performing method, by being able to improve both Magnesium and Gas Pockets IoU score, similar to Table 1a, thus also resulting in a higher mean IoU.

On the other hand, in both Table 1b, c, HMM-TC is only able to improve the IoU score of Gas Pocket regions, and not Magnesium. Therefore, in this case HMM-TC is not always better than unrefined segmentations. Alternatively, a basic median filter over time may offer similar or slightly better results than the original Stacked-DenseUSeg’s segmentations. The particularly high accuracy of HMM-T, however, may have occurred due to the use of its predictions as a template for the annotations of the ground-truth used in Table 1b, c, potentially creating a high correlation between them. The influence of this correlation could also explain the better performance of HMM-T-naive in Table 1b and HMM-T-naive-stable in Table 1b, c (both of which share the same observation matrix with HMM-T) over HMM-TC, dissimilar to Table 1a. Sadly, attempting to manually segment using Stacked-DenseUSeg’s prediction, or worse, without any segmentation labels as a guide, would be extremely difficult for the annotator. Despite best manual efforts, we cannot overlook this as a potential factor in the dynamic regions, presented in Table 1b, c. Nonetheless, the fact that HMM-T predictions provide an excellent starting point, as verified by close manual inspection during the annotation refinement, is itself an argument for its accuracy. Also to note, both naive and naive-stable versions do not outperform HMM-T overall despite deriving from this model. Furthermore, on visual inspection, HMM-TC does not display signs of misbehaviour for input predictions that include temporal changes. In Fig. 5b, for example, mark $\varvec{L_5}$ points out that that HMM-TC is able to approach the manual annotations for the difficult features identified by the marks $\varvec{L_1}$–$\varvec{L_4}$.

**Table 1:** Intersection over Union (IoU) segmentation results using: (**a**) using as ground-truth the annotations from the representative to the time-series fully-sampled tomogram, (**b**) and (**c**) using as ground-truth our manual 4D annotations capturing state changes.

	IoU of Air	IoU of Aluminium	IoU of Magnesium	IoU of Gas Pockets	Mean IoU
(**a**) Tested in an area without temporal events (for 10 slices over time, $10{\times }21{=}210$ images)					
Unrefined predictions of Stacked-DenseUSeg	0.987	0.966	0.405	0.062	0.605
HMM-T	**0.990**	0.967	**0.431**	**0.122**	**0.628**
HMM-TC	**0.990**	0.967	0.430	0.085	0.618
HMM-T-naive	0.987	0.966	0.405	0.062	0.605
HMM-T-naive-stable	0.987	0.966	0.42	0.078	0.613
Median Filter (1D filter with window size of 5)	0.989	**0.968**	0.412	0.052	0.605
(**b**) Tested in an area with temporal events (for 3 contiguous slices over time, $3{\times }21{=}63$ images)					
Unrefined predictions of Stacked-DenseUSeg	0.986	0.987	0.74	0.237	0.738
HMM-T	**0.991**	**0.995**	**0.957**	**0.341**	**0.821**
HMM-TC	0.989	0.986	0.687	0.24	0.725
HMM-T-naive	0.986	0.987	0.74	0.237	0.738
HMM-T-naive-stable	0.986	0.989	0.823	0.271	0.767
Median Filter (1D filter with window size of 5)	0.989	0.99	0.792	0.23	0.75
(**c**) Tested in an area with temporal events (for 3 contiguous slices over time, $3{\times }21{=}63$ images)					
Unrefined predictions of Stacked-DenseUSeg	0.988	0.988	0.757	0.202	0.734
HMM-T	**0.993**	**0.996**	**0.967**	**0.492**	**0.862**
HMM-TC	0.99	0.987	0.721	0.32	0.755
HMM-T-naive	0.988	0.988	0.757	0.202	0.734
HMM-T-naive-stable	0.988	0.99	0.829	0.241	0.762
Median Filter (1D filter with window size of 5)	0.99	0.99	0.802	0.152	0.734

In (**a**) the comparisons are performed in an area of the sample where temporal events are minimal, for 10 slices (slices from height 1210–1220) and through 21 tomogram time-series ($10{\times }21{=}210$ images). In (**b**) and (**c**) the comparisons are in areas where temporal events likely occurred. In (**b**) the comparisons are for 3 contiguous slices (slices from height 740–742) and through 21 tomogram time-series ($3{\times }21{=}63$ images) and in (**c**) the comparisons are for another 3 contiguous slices (slices from height 747–749) and through time ($3{\times }21{=}63$ images). The presented approaches all refine Stacked-DenseUSeg’s segmentation predictions of the tomogram time-series, which were obtained by applying Stacked-DenseUSeg to each of tomogram of the time-series independently. These methods as presented in “Methods” section are the HMM-T, HMM-TC, the HMM-T-naive, the HMM-T-naive-stable and a 1D median filter with window size of 5 time points. The latter three of these models are used as a baseline against our proposed HMM-T and HMM-TC. In (**a**) HMM-T and HMM-TC models overall improve the accuracy of Stacked-DenseUSeg’s segmentation predictions better than the HMM-T-naive, HMM-T-naive-stable and the median filter. In (**b**) and (**c**), only HMM-T model improves Stacked-DenseUSeg’s segmentation predictions consistently better than the HMM-T-naive, HMM-T-naive-stable and the median filter. The bold value(s) in each column signify the corresponding approach achieved the best IoU score for respective column.

## Discussion

In conclusion, we have presented here a novel use of HMMs for the temporal refinement of multiple tomogram segmentations that derive from a time-series of tomograms. Namely, we have presented two models, HMM-T and HMM-TC, that each receive as input 4D segmentation predictions generated by multiple individual 3D CNN outputs, independent of time. Our models refine these based on the expected transition probabilities of the classes through time. Additionally, they use the 3D CNN’s^11^ confusion matrix to help determine their emission probability matrices. Moreover with HMM-TC, in addition to the CNN’s confusion matrix, this model also uses the probabilities ascribed by the CNN to its predictions, to try and handle ambiguous labels. This was done by expanding its observation classes to include low, medium and high confidence versions of each class.

It can be seen in “Qualitative assessment of the HMM predictions” section, that the quality of the output 4D segmentation labels after the use of our models is often better compared to the initial CNN segmentations applied independently through time. The most profound outcome is the tidying of class transitions in “edge-toxels”, due to noise (see Fig. 3). Moreover, during behavioural experiments, HMM-T displayed to be able to halt unexpected transitions, when they appear “noise-like” (See the left-hand side of Fig. 4). The existence of multiple observations, contrary to the expectations, over time and the high emission probabilities of these eventually allow improbable transitions (See Figure right-hand side of Fig. 4). This suggests that our models are suitable for the refinement of 4D segmentation labels, as they can fix temporally incoherent segmentations caused by misclassification from the Stacked-DenseUSeg, but they still allow physically viable temporal changes to manifest in the segmentations of the time-series’s tomograms.

Finally, we measured the relative accuracy of the segmentation itself after the use of our models. In static areas (ie. with no temporal events), both models are able to remove temporal incoherence and improve segmentation, with HMM-T being the best performing model in general. HMM-T also performs better in areas where temporal events likely occurred. However we report this with some reservations, as noted earlier, due to the potential high correlation between the manual annotations used and the HMM-T predictions. This is an inherent limitation of our approaches, since accurate evaluation of our HMMs’ refinement in dynamically-changing regions is impossible without accurate 4D annotations. Due to the extreme low SNR of the time-series’ tomogram reconstructions, accurate manual annotations are almost impossible to obtain. This prompted us to use HMM-T’s predictions as template for our manual annotations, since a satisfactory manual annotation without any pre-existing aid would require several weeks of manual labor. Still, the value of our approach is shown in static regions (using accurate annotations derived from the highly-sampled representative tomogram) in the qualitative and behavioural experiments. Furthermore, as mentioned in the Introduction, due to noisy input data and the lack of accurate 4D annotated data, our HMMs cannot be compared against other state-of-the-art 4D segmentation approaches. Moreover, comparing against them would not constitute a valid comparison, since our HMMs are designed to only temporally refine pre-made segmentations (see Fig. 2c). For these reasons, they are instead compared against more simplistic models: HMM-T-naive, HMM-T-naive-stable and a median filter (see “Selection of hidden Markov model parameters for the segmentation of time-series of tomogram reconstructions” section). From these experiments we can observer HMM-T’s overall best performance, which could derive from the transition matrix determined based on the expected behaviour for the physical system.

HMM-TC does not show the same level of accuracy in terms of IoU, however in Fig. 5 its outputs closely resemble ground-truth features. The visual quality of its refinements is similar to the HMM-T, to the degree that we could also may had used them as a template for our manual annotations. Its main measurable drawback is its poor refinement of Magnesium toxels, in dynamically-changing regions. The cause of this is not clear, but one hypothesis is that it is due to the manually-adjusted probabilities in HMM-TC’s observation matrix. These were adjusted to try compensate for “information-loss-caused” misclassifications, that are considered in Stacked-DenseUSeg’s confusion matrix (more in “Selection of hidden Markov model parameters for the segmentation of time-series of tomogram reconstructions” section. However, it seems clear the resultant table is still not optimal; these matrices are both the strength and weakness of HMM methods. In the future, an automated technique for selecting the optimal HMM-TC’s observation probabilities could be investigated. Lastly, an alternative HMM-TC configuration could be investigated, which does not utilize a traditional observation matrix. For example, this new configuration could use as observation probabilities for each toxel the class likelihood predicted by Stacked-DenseUSeg for the respective toxel.

Furthermore, it is important to state that both HMM-T and HMM-TC, are based on empirically determined transition probabilities. In the future, this may be resolved by ensuring the collection of at least two representative highly-sampled tomograms (like the one used to train the Stacked-DenseUSeg approach), one collected before the collection of the time-series of tomograms and one after. By performing this step, it would be possible to perform a post-comparison of the two highly-sampled tomograms, to accurately estimate the transition probabilities of non-reversible reactions. Additionally, it would allow the pipeline to utilize HMMs with different transition probabilities in different regions, further improving the accuracy of the refined segmentation. It seems clear to us that although we demonstrate the potential application of the HMM approach, much future work is possible in refining these parameters.

Concluding, the use of HMMs has allowed the refinement of multiple time-resolved 3D CNN segmentations obtained independently through time, without the use of 4D annotated data. Our proposed HMMs opens the road for future expansion of 3D CNNs for 4D data, especially in cases where 4D annotated data are not available.

## Supplementary Information

Supplementary Information 1.
