# Supplementary material for: Temporal refinement of 3D CNN semantic segmentations on 4D time-series of undersampled tomograms using hidden Markov models
Source: Sci Rep. 2021 Dec 2;11:23279. doi: 10.1038/s41598-021-02466-x (PMC8640015; doi:10.1038/s41598-021-02466-x)
Supplement: Supplementary file 1 — Supplementary Information 1. [file 41598_2021_2466_MOESM1_ESM.pdf]

Temporal refinement of 3D CNN semantic segmentations on 4D time-series of undersampled tomograms using hidden Markov models

Dimitrios Bellos, Andrew P. French, Mark Basham, Tony Pridmore

Supplementary Information

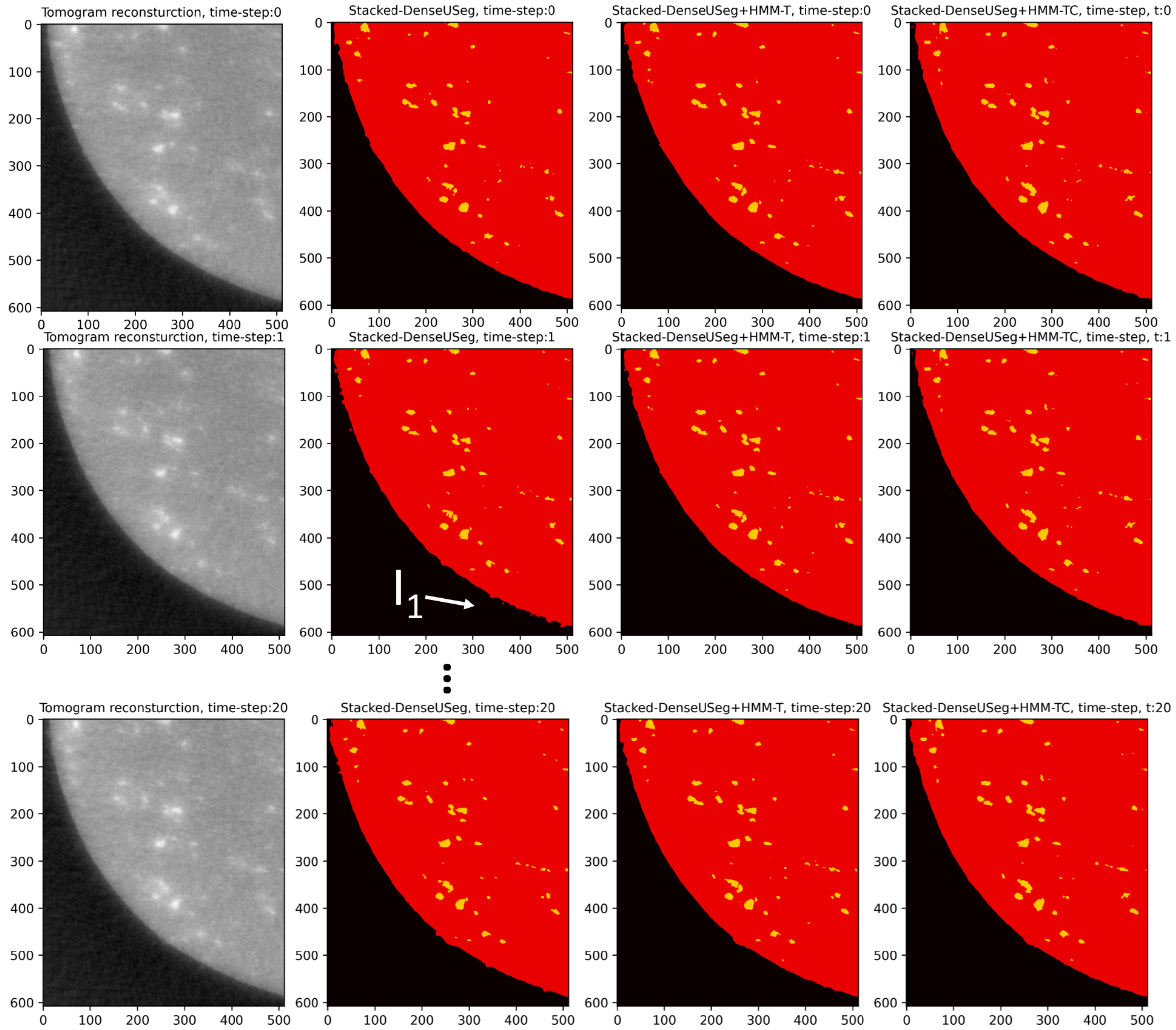

Supplementary Figure 1: *Segmentation predictions of a time-series of tomograms after the use of Stacked-DenseUSeg, Stacked-DenseUSeg + HMM-T, Stacked-DenseUSeg + HMM-TC. The predictions are a time-series of 21 tomograms for the slice of height* 755th *(out of 1710)* $(y - axis)$ *and for the ROI [696:1304, 10:522]* $(xz - plane)$*. As it can be seen from mark* $\boldsymbol{I_1}$*, the HMM models corrected the jagged edges of the Stacked-DenseUSeg predictions and in general the changes are very subtle, meaning that they work to refine the predictions.*

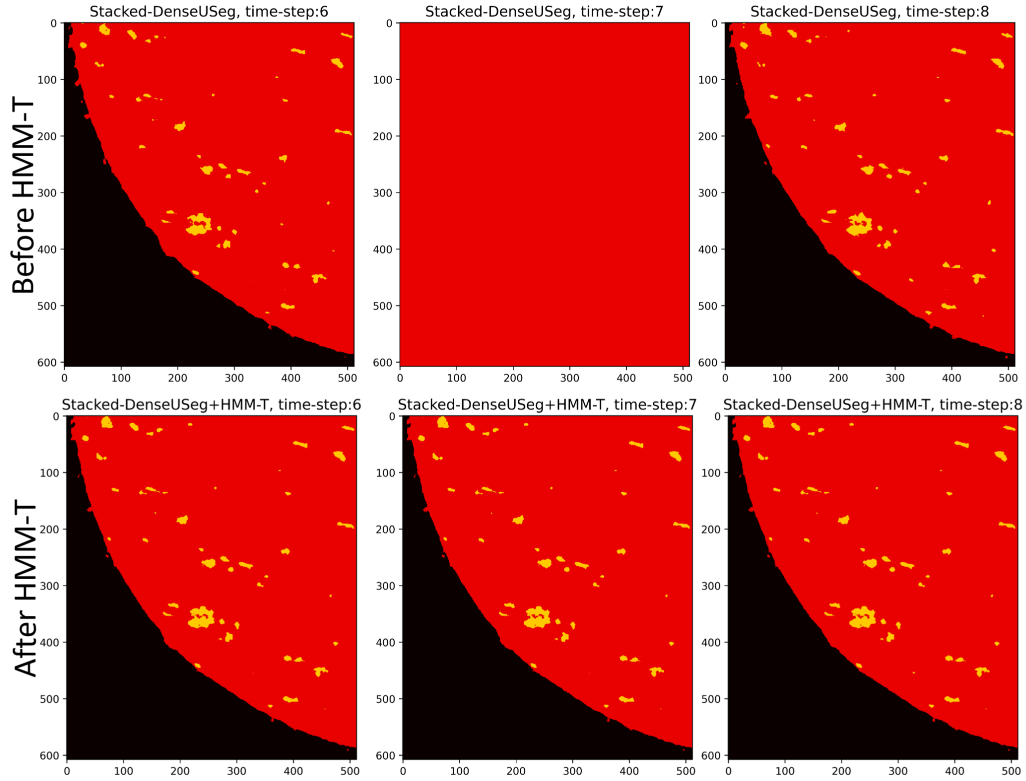

Supplementary Figure 2: *Behavioral test of the HMM-T for the prediction of the restoration of information using information from adjacent time-steps. For this test the HMM-T is being used. In this figure the predictions for the* 7th *tomogram in the time-series are being replaced completely with toxels that belong to the class of Aluminium (red). The slice displayed is the* 740th *(out of 1710) of the height dimension* $(y-axis)$*, for the ROI [696:1304, 10:522]* $(xz-plane)$*. As it can be seen through the use of adjacent time-steps even annotations for missing time-steps can be recovered with a sufficient level of accuracy*

$$\boldsymbol{T} = \begin{array}{c} \\ \\ \\ \\ \end{array} \begin{array}{cccc} Air & Aluminium & Magnesium & Gas\ Pockets \end{array} \\ \begin{bmatrix} 0.998 & 0.001 & 0.001 & 0 \\ 0.001 & 0.996 & 0.001 & 0.002 \\ 0.001 & 0.001 & 0.8982 & 0.0998 \\ 0 & 0.001 & 0.001 & 0.998 \end{bmatrix} \begin{array}{c} Air \\ Aluminium \\ Magnesium \\ Gas\ Pockets \end{array} \quad (1)$$

Supplementary Matrix 1: *The Transition Matrix for HMM-T and HMM-TC. It depicts the probability of each segmentation classes to transition to a different one through time, therefore they are hand-picked so that the sum of each row to equal to one*

$$\boldsymbol{T_{naive}} = \begin{array}{cccc} Air & Aluminium & Magnesium & Gas\ Pockets \end{array} \\ \begin{bmatrix} 0.25 & 0.25 & 0.25 & 0.25 \\ 0.25 & 0.25 & 0.25 & 0.25 \\ 0.25 & 0.25 & 0.25 & 0.25 \\ 0.25 & 0.25 & 0.25 & 0.25 \end{bmatrix} \begin{array}{c} Air \\ Aluminium \\ Magnesium \\ Gas\ Pockets \end{array} \quad (2)$$

Supplementary Matrix 2: *The Transition Matrix for the naive implementation of HMM-T (HMM-T-naive). It depicts the probability of each segmentation classes to transition to a different one through time. The probabilities are picked so that it is equally probable to transition from any class to any other class including its current one. By using the above the transition matrix, HMM-T-naive considers any input sequence of classes equally probable and therefore there is not a sequence of classes that it would consider containing temporal incoherence, that has to be resolved.*

$$\boldsymbol{T_{naive-stable}} = \begin{array}{c} \\ \\ \\ \\ \\ \end{array}\begin{array}{cccc} \mathit{Air} & \mathit{Aluminium} & \mathit{Magnesium} & \mathit{Gas\ Pockets} \\ \left[\begin{array}{c} 0.5 \\ 0.16666 \\ 0.16666 \\ 0.16666 \end{array}\right. & \begin{array}{c} 0.16666 \\ 0.5 \\ 0.16666 \\ 0.16666 \end{array} & \begin{array}{c} 0.16666 \\ 0.16666 \\ 0.5 \\ 0.16666 \end{array} & \left.\begin{array}{c} 0.16666 \\ 0.16666 \\ 0.16666 \\ 0.5 \end{array}\right] \end{array} \begin{array}{c} \\ \mathit{Air} \\ \mathit{Aluminium} \\ \mathit{Magnesium} \\ \mathit{Gas\ Pockets} \end{array} \quad (3)$$

Supplementary Matrix 3: *The Transition Matrix for the naive-stable implementation of HMM-T (HMM-T-naive-stable). It depicts the probability of each segmentation classes to transition to a different one through time. The probabilities are picked so that it is 50% probable that a class will not change in the next time-step and 50% that it will and if so, it has equal probability to change to any other class excluding its current one. By using the above the transition matrix, HMM-T-naive-stable can refine input sequences of classes that have multiple consecutive transitions to different classes than the current one, (e.g sequences like Air, Aluminium, Magnesium, Air, Gas Pockets, Magnesium and so on) slightly better than HMM-T-naive. However, apart from the information that it is slightly more probable to have input sequences of classes that do not have multiple consecutive transitions, it does not utilise any special information about the physical system depicted in the 4D dataset. For example, that in corrosion experiments like the one depicted in 4D dataset used, it is a lot less probable for classes to transition to different ones through time, and when they do, that it is more probable Magnesium class voxels to transition to Gas Pocket class voxels due to the undergoing corrosion.*

$$\boldsymbol{C_1} = \begin{array}{cccc} \mathit{Predicted} & \mathit{Predicted} & \mathit{Predicted} & \mathit{Predicted} \\ \mathit{Air} & \mathit{Aluminium} & \mathit{Magnesium} & \mathit{Gas\ Pockets} \\ \left[\begin{array}{c} 27171100 \\ 148082 \\ 115 \\ 10 \end{array}\right. & \begin{array}{c} 265330 \\ 239760000 \\ 4118587 \\ 32301 \end{array} & \begin{array}{c} 35 \\ 1747349 \\ 5080109 \\ 227 \end{array} & \left.\begin{array}{c} 4 \\ 18310 \\ 633 \\ 52233 \end{array}\right] \end{array} \begin{array}{c} \\ \\ \mathit{Air} \\ \mathit{Aluminium} \\ \mathit{Magnesium} \\ \mathit{Gas\ Pockets} \end{array} \quad (4)$$

Supplementary Matrix 4: *The Confusion Matrix which the Emission Matrix of HMM-T is based upon. It depicts the the number of instances that each voxel in the testing dataset of Stacked-DenseUSeg was predicted correctly or incorrectly as a different class.*

$$\boldsymbol{O_1} = \begin{array}{cccc} \mathit{Predicted} & \mathit{Predicted} & \mathit{Predicted} & \mathit{Predicted} \\ \mathit{Air} & \mathit{Aluminium} & \mathit{Magnesium} & \mathit{Gas\ Pockets} \\ \left[\begin{array}{c} \mathbf{9.946 \cdot 10^{-1}} \\ 1.087 \cdot 10^{-3} \\ 5.126 \cdot 10^{-6} \\ 5.620 \cdot 10^{-5} \end{array}\right. & \begin{array}{c} 5.420 \cdot 10^{-3} \\ \mathbf{9.819 \cdot 10^{-1}} \\ 2.559 \cdot 10^{-1} \\ 2.572 \cdot 10^{-1} \end{array} & \begin{array}{c} 4.209 \cdot 10^{-6} \\ 1.687 \cdot 10^{-2} \\ \mathbf{7.440 \cdot 10^{-1}} \\ 8.893 \cdot 10^{-3} \end{array} & \left.\begin{array}{c} 3.660 \cdot 10^{-7} \\ 1.323 \cdot 10^{-4} \\ 3.325 \cdot 10^{-5} \\ \mathbf{7.338 \cdot 10^{-1}} \end{array}\right] \end{array} \begin{array}{c} \\ \\ \mathit{Air} \\ \mathit{Aluminium} \\ \mathit{Magnesium} \\ \mathit{Gas\ Pockets} \end{array} \quad (5)$$

Supplementary Matrix 5: *The Emission Matrix for HMM-T. It depicts the probability of each 3D CNN predicted classes to be each of the final segmentation predictions.*

$C_2 =$

Low − Conf Air	*Mid − Conf Air*	*High − Conf Air*	*Low − Conf Aluminium*	*Mid − Conf Aluminium*	*High − Conf Aluminium*	
615	58203	27112284	650	45731	101700	*Air*
1055	64192	200083	5503	1001348	238755654	*Aluminium*
5	23	7	998	207744	1538605	*Magnesium*
2	1	1	63	2552	15695	*Gas Pockets*

Low − Conf Magnesium	*Mid − Conf Magnesium*	*High − Conf Magnesium*	*Low − Conf Gas Pockets*	*Mid − Conf Gas Pockets*	*High − Conf Gas Pockets*	
17	55	43	2	7	1	*Air*
4407	910667	3203510	134	5614	26553	*Aluminium*
1016	269887	4809208	8	74	145	*Magnesium*
26	235	372	42	2354	49837	*Gas Pockets*

(6)

Supplementary Matrix 6: *The Confusion Matrix which the Emission Matrix of HMM-TC is based upon. It depicts the the number of instances that each voxel in the testing dataset of Stacked-DenseUSeg was predicted correctly (and how certain was the network about the prediction) or incorrectly as a different class (and again how certain was the network about the prediction).*

$O_2 =$

Low − Conf Air	*Mid − Conf Air*	*High − Conf Air*	*Low − Conf Aluminium*	*Mid − Conf Aluminium*	*High − Conf Aluminium*	
$2.251 \cdot 10^{-5}$	$\mathbf{2.130 \cdot 10^{-3}}$	$\mathbf{9.924 \cdot 10^{-1}}$	$2.379 \cdot 10^{-5}$	$1.674 \cdot 10^{-3}$	$3.723 \cdot 10^{-3}$	*Air*
$4.321 \cdot 10^{-6}$	$2.629 \cdot 10^{-4}$	$8.194 \cdot 10^{-4}$	$2.254 \cdot 10^{-5}$	$4.101 \cdot 10^{-3}$	$\mathbf{9.778 \cdot 10^{-1}}$	*Aluminium*
$7.323 \cdot 10^{-7}$	$3.369 \cdot 10^{-6}$	$1.025 \cdot 10^{-6}$	$1.462 \cdot 10^{-4}$	$3.043 \cdot 10^{-2}$	$2.253 \cdot 10^{-1}$	*Magnesium*
$\mathbf{2.810 \cdot 10^{-5}}$	$1.405 \cdot 10^{-5}$	$1.405 \cdot 10^{-5}$	$\mathbf{8.851 \cdot 10^{-4}}$	$\mathbf{3.585 \cdot 10^{-2}}$	$2.205 \cdot 10^{-1}$	*Gas Pockets*

Low − Conf Magnesium	*Mid − Conf Magnesium*	*High − Conf Magnesium*	*Low − Conf Gas Pockets*	*Mid − Conf Gas Pockets*	*High − Conf Gas Pockets*	
$6.223 \cdot 10^{-7}$	$2.013 \cdot 10^{-6}$	$1.574 \cdot 10^{-6}$	$7.321 \cdot 10^{-8}$	$2.562 \cdot 10^{-7}$	$3.660 \cdot 10^{-8}$	*Air*
$1.805 \cdot 10^{-5}$	$3.730 \cdot 10^{-3}$	$1.312 \cdot 10^{-2}$	$5.488 \cdot 10^{-7}$	$2.299 \cdot 10^{-5}$	$1.087 \cdot 10^{-4}$	*Aluminium*
$1.488 \cdot 10^{-4}$	$\mathbf{3.953 \cdot 10^{-2}}$	$\mathbf{7.044 \cdot 10^{-1}}$	$1.172 \cdot 10^{-6}$	$1.084 \cdot 10^{-5}$	$2.124 \cdot 10^{-5}$	*Magnesium*
$\mathbf{3.653 \cdot 10^{-4}}$	$3.301 \cdot 10^{-3}$	$5.226 \cdot 10^{-3}$	$\mathbf{5.901 \cdot 10^{-4}}$	$\mathbf{3.307 \cdot 10^{-2}}$	$\mathbf{7.002 \cdot 10^{-1}}$	*Gas Pockets*

(7)

Supplementary Matrix 7: *The original Emission Matrix for HMM-TC. It depicts the probability of each 3D CNN predicted classes (also split to 3 confidence "bins") to be each of the final segmentation predictions. As it can be seen for the observable states, sometimes they are emitted with higher probability by classes that are not the leading class.*

$$
\boldsymbol{O}_2^* =
\begin{array}{c}
\begin{array}{cccccc}
Low-Conf & Mid-Conf & High-Conf & Low-Conf & Mid-Conf & High-Conf \\
Air & Air & Air & Aluminium & Aluminium & Aluminium
\end{array} \\
\left[\begin{array}{cccccc|c}
\mathbf{2.251 \cdot 10^{-5}} & \mathbf{2.130 \cdot 10^{-3}} & \mathbf{9.924 \cdot 10^{-1}} & 2.141 \cdot 10^{-5} & 1.674 \cdot 10^{-3} & 3.723 \cdot 10^{-3} & Air \\
4.321 \cdot 10^{-6} & 2.629 \cdot 10^{-4} & 8.194 \cdot 10^{-4} & \mathbf{2.254 \cdot 10^{-5}} & \mathbf{4.101 \cdot 10^{-3}} & \mathbf{9.778 \cdot 10^{-1}} & Aluminium \\
7.323 \cdot 10^{-7} & 3.369 \cdot 10^{-6} & 1.025 \cdot 10^{-6} & 2.193 \cdot 10^{-5} & 4.077 \cdot 10^{-3} & 2.253 \cdot 10^{-1} & Magnesium \\
2.810 \cdot 10^{-7} & 1.405 \cdot 10^{-5} & 1.405 \cdot 10^{-7} & 8.851 \cdot 10^{-6} & 3.585 \cdot 10^{-4} & 2.205 \cdot 10^{-2} & Gas\ Pockets \\
 & & & & & & \\
Low-Conf & Mid-Conf & High-Conf & Low-Conf & Mid-Conf & High-Conf & \\
Magnesium & Magnesium & Magnesium & Gas\ Pockets & Gas\ Pockets & Gas\ Pockets & \\
6.223 \cdot 10^{-7} & 2.013 \cdot 10^{-6} & 1.574 \cdot 10^{-6} & 7.321 \cdot 10^{-8} & 2.562 \cdot 10^{-7} & 3.660 \cdot 10^{-8} & Air \\
1.805 \cdot 10^{-5} & 3.730 \cdot 10^{-3} & 1.312 \cdot 10^{-2} & 5.488 \cdot 10^{-7} & 2.299 \cdot 10^{-5} & 1.087 \cdot 10^{-4} & Aluminium \\
\mathbf{1.488 \cdot 10^{-4}} & \mathbf{3.953 \cdot 10^{-2}} & \mathbf{7.044 \cdot 10^{-1}} & 1.172 \cdot 10^{-6} & 1.084 \cdot 10^{-5} & 2.124 \cdot 10^{-5} & Magnesium \\
7.305 \cdot 10^{-7} & 3.301 \cdot 10^{-3} & 5.226 \cdot 10^{-3} & \mathbf{5.901 \cdot 10^{-4}} & \mathbf{3.307 \cdot 10^{-2}} & \mathbf{7.002 \cdot 10^{-1}} & Gas\ Pockets
\end{array}\right]
\end{array}
\quad (8)
$$

Supplementary Matrix 8: *The final Emission Matrix for HMM-TC. It depicts the probability of each 3D CNN predicted classes (also split to 3 confidence "bins") to be each of the final segmentation predictions. As it can be seen now its observable class is emitted with higher probability always by the leading class.*
